# Posttraumatic stress disorder is associated with an enhanced spontaneous production of pro-inflammatory cytokines by peripheral blood mononuclear cells

**DOI:** 10.1186/1471-244X-13-40

**Published:** 2013-01-29

**Authors:** Hannah Gola, Harald Engler, Annette Sommershof, Hannah Adenauer, Stephan Kolassa, Manfred Schedlowski, Marcus Groettrup, Thomas Elbert, Iris-Tatjana Kolassa

**Affiliations:** 1 Clinical Psychology & Neuropsychology, University of Konstanz, Konstanz, Germany; 2 Institute of Medical Psychology and Behavioral Immunobiology, University Hospital Essen, University of Duisburg-Essen, Essen, Germany; 3 Division of Immunology, University of Konstanz, Konstanz, Germany; 4 Research & Innovation, Center of Excellence Forecasting & Replenishment, SAP AG, Tägerwilen, Switzerland; 5 Clinical & Biological Psychology, Institute of Psychology & Education, University of Ulm, Albert-Einstein-Allee 47, 89069, Ulm, Germany

**Keywords:** Posttraumatic stress disorder, Immune system, Cytokines, Pro-inflammatory cytokines, Traumatic stress, Inflammation

## Abstract

**Background:**

Posttraumatic stress disorder (PTSD) is associated with an enhanced risk for cardiovascular and other inflammatory diseases. Chronic low-level inflammation has been suggested as a potential mechanism linking these conditions.

**Methods:**

We investigated plasma cytokine levels as well as spontaneous and lipopolysaccharide (LPS)-stimulated cytokine production by peripheral blood mononuclear cells (PBMCs) in a group of 35 severely traumatized PTSD patients compared to 25 healthy controls.

**Results:**

Spontaneous production of interleukin (IL)-1β, IL-6 and tumor necrosis factor (TNF)-α by isolated PBMCs was significantly higher in the PTSD compared to the control group and even correlated with PTSD symptom severity within the PTSD group. In contrast, circulating plasma levels of pro- and anti-inflammatory cytokines such as IL-6, IL-8, IL-10, TNF-α, or monocyte chemotactic protein (MCP)-1 were not significantly altered in PTSD patients compared to healthy controls.

**Conclusions:**

Our findings indicate that PBMCs of PTSD patients are already pre-activated *in vivo*, providing further evidence for low-grade inflammation in PTSD. This might possibly represent one psychobiological pathway from PTSD to poor physical health.

## Background

Repeated exposure to traumatic events such as rape, combat or natural disasters has dramatic effects on mental health [1-3]. Frequent symptoms in the aftermath of such events include repetitive intrusive recollections of the trauma, persistent avoidance of trauma reminders, emotional numbing and hyperarousal – the core symptoms of posttraumatic stress disorder PTSD [4].

In addition to psychiatric morbidity, numerous studies have shown that traumatic stress and especially PTSD are associated with poor self-reported physical health, increased utilization of medical services, and an elevated risk for multiple comorbid medical disorders such as respiratory, gastrointestinal, musculoskeletal, inflammatory and autoimmune diseases [5-7]. In particular, it has been found that cardiovascular disease and its risk factors are more prevalent among individuals with PTSD [5,6,8], with two recent studies even demonstrating a prospective relationship between PTSD symptoms and coronary heart disease [9] or PTSD and cardiovascular mortality, respectively [10].

Besides traditional behavioral cardiovascular risk factors such as smoking, alcohol abuse or low physical activity [11], chronic low-level inflammation has been discussed as a potential mechanism linking cardiovascular disease to PTSD [12]. This argumentation is supported by epidemiological and clinical studies demonstrating a strong and consistent relationship between markers of inflammation and risk of future cardiovascular events [13,14].

However, studies investigating pro-inflammatory markers in PTSD patients have yielded mixed results. Some studies reported higher levels of IL-1β [15,16], IL-6 [17], and TNF-α [12] in the plasma or elevated levels of IL-6 in the cerebrospinal fluid [18] of PTSD patients compared to control subjects. Furthermore, Spitzer et al. [19] found that, in a sample of 3049 adults, PTSD positive participants had significantly higher odds for elevated levels of C-reactive protein (CRP) than those without PTSD. In contrast, other studies did not find significant group differences with respect to circulating levels of IL-1β [12], IL-6 [12,20], and CRP [12] or even reported lower levels of CRP [21] and IL-8 [20] in PTSD patients.

Likewise, ambiguous results have been obtained by studies investigating the production of pro-inflammatory cytokines in whole blood following *ex vivo* stimulation with lipopolysaccharide (LPS) and/or phytohemagglutinin (PHA). Whereas some studies reported higher LPS-stimulated IL-6 production [22] or increased LPS/PHA-induced production of IL-6 and TNF-α [23] in individuals with PTSD, other studies showed no group differences with respect to PHA-induced interferon (IFN)-γ [24] or LPS-induced TNF-α production [22]. One study by de Kloet et al. [24] even reported decreased TNF-α production in LPS-stimulated whole blood of PTSD patients compared to controls.

These inconsistent findings might partly be explained by variations in sample characteristics, such as differences with respect to the type of traumata experienced (e.g. childhood vs. adulthood trauma), time elapsed since the traumata (e.g. populations studied shortly after traumatic experiences or during ongoing threat vs. patients with chronic symptoms), comorbidities, and differences with respect to PTSD symptom severity (mild symptoms after a single trauma in comparison to severe symptoms after experiencing multiple traumata), as well as varying sample sizes. In particular studies investigating plasma cytokine levels consisted mostly of small patient groups, making the results error-prone.

Here we investigated plasma levels of pro- and anti-inflammatory cytokines in a well-defined group of 35 severely affected PTSD patients with war and torture experiences mainly experienced during late adolescence and adulthood and a chronic disease pattern, compared to 25 healthy ethnically matched control subjects. For a subsample of 16 PTSD patients and 18 control subjects, we additionally analyzed 1) LPS-stimulated production of IL-1β, IL-6 and TNF-α by peripheral blood mononuclear cells (PBMCs), as well as 2.) spontaneous cytokine production by these cells, as this has not been reported previously.

## Methods

### Participants

We analyzed 35 individuals with current PTSD (19 male, 16 female; mean age = 32.6, range 16–51) according to the DSM-IV [4] and 25 healthy control subjects (8 male, 17 female; mean age = 26.8 years, range 18–45). PTSD patients were refugees, with chronic (mean symptom duration = 7.6 years, SD = 4.6) and severe (mean sum score in the *Clinician Administered PTSD Scale*, CAPS [25] = 80.0, SD = 17.5) forms of PTSD due to multiple highly stressful war and torture experiences, mainly experienced during late adolescence and adulthood. In addition to the PTSD diagnosis, 27 patients met the DSM-IV criteria for a current major depressive episode. Thirteen PTSD patients reported current intake of hypnotic, anxiolytic, antidepressant or neuroleptic medication and one woman reported the use of oral contraceptives. Thirty-seven percent of the PTSD patients were smokers (for subjects’ characteristics see Table 1). All patients were recruited from the Psychotrauma Research and Outpatient Clinic for Refugees, University of Konstanz, Germany. The healthy control group was recruited through advertisement and was comparable to the patient group with regard to region of origin. Except for four women reporting the intake of oral contraceptives, all control subjects were free of medication. Sixteen percent of the control subjects were smokers (see Table 1).

**Table 1:** Sociodemographic and clinical characteristics of PTSD patients and controls

**Variables**	**PTSD**	**Controls**	***p***
	**(n = 35)**	**(n = 25)**	
Age^a^ (y)	32.6 ± 9.1	29.1 ± 9.7	.01
Sex (f/m)	16/19	17/8	.09
Region of Origin (%)			.06
*Africa*	*20.0%*	*8.0%*	
*Balkan*	*17.2%*	*48.0%*	
*Middle East*	*62.8%*	*44.0%*	
Smokers (%)	37.1%	16.0%	.07
Psychotropic medication (%)	37.1%	0%	<.0005
*Hypnotics*	*14.3%*	*0%*	
*Anxiolytics*	*11.4%*	*0%*	
*Antidepressives*	*25.7%*	*0%*	
*Neuroleptics*	*8.6%*	*0%*	
Contraceptives (%)	2.8%	16.0%	.10
Comorbid depression (%)	77.1%	0%	<.0005
Number of traumatic event types^a^			
*War or torture events*	*10.8 ± 5.4*	*2.3 ± 5.0*	*<.0005*
*CAPS events*	*6.9 ± 2.1*	*3.4 ± 2.0*	*<.0005*
CAPS Score^a^	80.0 ± 17.5	5.4 ± 11.2	<.0005
HAMD Score^a^	26.1 ± 8.2	4.1 ± 5.6	<.0005
SOMS Score^a^	28.7 ± 12.4	5.5 ± 8.5	<.0005

^a^Data are presented as mean ± SD.

*For pair-wise group comparisons of continuous variables, we performed *t*-tests, differences of categorical variables were evaluated by applying χ^2^ tests for independence. (Y) = years; (f) = female; (m) = male; CAPS, Clinician Administered PTSD Scale; HAM-D, Hamilton Depression Rating Scale; SOMS-7, Screening for Somatoform Symptoms-7.

Exclusion criteria for the study were intake of glucocorticoids or acute (1 PTSD patient and 2 controls) and chronic (1 PTSD patient and 3 controls) somatic illnesses. In addition, control subjects were excluded if they met the criteria for any mental disorder according to DSM-IV (n = 4), or reported intake of psychotropic medication (n = 2). PTSD patients were excluded if they met the criteria for comorbid alcohol or substance abuse and dependence (n = 3) or a current or past history of a psychosis (n = 1) according to DSM-IV. Furthermore, participants were screened for possible HIV and hepatitis A, B and C infections. All samples were negative for HIV or hepatitis C. Subjects classified with acute or chronic hepatitis A or B (3 PTSD patients and 3 controls) were excluded from the study, reducing the initially enrolled sample of 44 individuals with PTSD and 39 controls to 35 PTSD patients and 25 control participants.

Basal plasma cytokine measurements were obtained from all subjects (PTSD, n = 35; healthy controls, n = 25). Spontaneous and LPS-induced *cytokine production* by cultured *PBMCs* was investigated in a subsample of 16 PTSD patients and 18 control subjects (see Table 2 for the respective subject characteristics).

**Table 2:** Sociodemographic and clinical characteristics of the subgroup of PTSD patients and control subjects for which we analyzed cytokine production by PBMCs

**Variables**	**PTSD**	**Controls**	***p***
	**(n = 16)**	**(n = 18)**	
Age^a^ (y)	36.1 ± 9.5	26.7 ± 7.4	.003
Sex (f/m)	7/9	11/7	.31
Region of Origin (%)			.16
*Africa*	*18.7%*	*5.6%*	
*Balkan*	*25.0%*	*55.5%*	
*Middle East*	*65.3%*	*38.9%*	
Smokers (%)	37.5%	16.7%	.17
Psychotropic medication (%)	31.2%	0%	.01
*Hypnotics*	*0%*	*0%*	
*Anxiolytics*	*12.5%*	*0%*	
*Antidepressives*	*31.2%*	*0%*	
*Neuroleptics*	*6.2%*	*0%*	
Contraceptives (%)	0%	16.7%	.23
Comorbid depression (%)	75.0%	0%	<.0005
Number of traumatic event types^a^			
*War or torture events*	*11.0 ± 5.8*	*1.8 ± 3.9*	*<.0005*
*CAPS events*	*7.2 ± 1.7*	*3.3 ± 1.9*	*<.0005*
CAPS Score^a^	82.5 ± 16.5	7.0 ± 12.8	<.0005
HAMD Score^a^	25.2 ± 8.1	4.6 ± 6.3	<.0005
SOMS Score^a^	28.4 ± 13.8	4.9 ± 8.2	<.0005

^a^Data are presented as mean ± SD.

*For pair-wise group comparisons of continuous variables, we performed *t*-tests, differences of categorical variables were evaluated by applying *χ*^*2*^ tests for independence. (Y) = years; (f) = female; (m) = male; CAPS, Clinician Administered PTSD Scale; HAM-D, Hamilton Depression Rating Scale; SOMS-7, Screening for Somatoform Symptoms-7.

### Procedure

All participants underwent an extensive standardized clinical interview administered by experienced clinical psychologists and trained translators, starting always at 10:00 a.m.: Upon arrival at the outpatient clinic, procedures were explained to the participants and written informed consent was obtained. Subsequently, blood (t_1_) for white blood cell (WBC) differential counts, plasma cytokines, and PBMC isolation was drawn. Afterwards, sociodemographic information as well as medical information was acquired. Somatic symptoms were assessed using a shortened version of *Screening for Somatoform Symptoms7* SOMS-7, [26]. During the second part of the interview, participants were interviewed in a standardized manner about their individual traumatic experiences using the event checklist of the CAPS [25] and the *vivo checklist of war*, *detention and torture events*[27], which assesses common traumatic experiences in conflict regions and during torture. Subsequently, PTSD symptom frequency and severity were assessed with the CAPS [25]. Finally, the *Mini International Neuropsychiatric Interview* M.I.N.I., [28] was applied to screen for potential comorbid mental disorders. In addition, depressive symptoms were assessed with the *Hamilton Depression Rating Scale* HAM-D, [29]. One week after the standardized clinical interview the participants were invited for a second time. Again, blood samples for plasma cytokines were collected at 10:00 am (t_2_), to test for the stability of basal (t_1_) cytokine levels.

All procedures were approved by the Ethics Committee of the University of Konstanz and were carried out in accordance with the Declaration of Helsinki 2008.

### WBC differential count

Absolute numbers of leukocytes, lymphocytes, neutrophils, monocytes, eosinophils, and basophils were obtained using an automated hematology analyzer (XT-2000i, Sysmex, Horgen, Switzerland).

### Cell culture

Peripheral blood mononuclear cells (PBMCs) were isolated from citrated blood by density gradient centrifugation using commercially available cell preparation tubes (*Vacutainer*® CPT™ Cell Preparation Tube, BD Biosciences, Franklin Lakes, NY, USA) according to the manufacturer’s instructions. Freshly isolated PBMCs (2 × 10^5^) were suspended in 200 μl RPMI 1640 containing 10% fetal bovine serum and were cultured for 24 h in 96-well flat-bottom culture plates in presence or absence of 1 μg/ml lipopolysaccharide (LPS) from *Salmonella abortus equi* (Sigma-Aldrich, Taufkirchen, Germany).

### Cytokine analysis

Cytokine levels in plasma and culture supernatants were quantified using multiplex bead-based assays (Bio-Plex Cytokine Assays, Bio-Rad Laboratories, Hercules, CA, USA). Samples were prepared according to the manufacturer’s instructions and were analyzed in triplicate on a dual-laser flow cytometer equipped with high throughput sampler (LSR II, BD Immunocytometry Systems, San Jose, CA, USA). Absolute cytokine levels were calculated based on the mean fluorescence intensity of cytokine standards. Detection limits of the assays, defined as the mean of background value plus 3 SD, were 0.07 pg/ml (IL-6), 0.75 pg/ml (IL-8), 0.14 pg/ml (IL-10), 0.24 pg/ml (TNF-α), and 0.38 pg/ml (MCP-1), respectively.

### Statistical analyses

Data analysis was performed using R 2.15.2 [30]. Group comparisons with respect to sociodemographic and clinical variables were performed using chi-square tests for categorical data and *t*-tests for continuous data. Basal plasma and LPS-stimulated cytokine levels were analyzed using ANOVAs with group (PTSD patients vs. controls) as independent variable. Since some of the immunological variables did not met requirements for parametric testing (normality of residuals), statistical significance for the immune measures was assessed by nonparametric permutation tests. In each case, the full model and a reduced model omitting the factor(s) of interest were fitted and the statistic of interest (usually an *F* statistic) was calculated. Next, residuals under the reduced model were randomly permuted 10,000 times. In each case, the randomly permuted residuals were added back to the (non-permuted) fitted values under the reduced model. The resulting randomized dependent values were then again used in fitting full and reduced models, yielding a “permutation” statistic. The *p* values reported below are given by the position of the original statistic in the empirical distribution of the permutation statistic [31,32]. In each case, we also investigated gender as well as smoking status as a covariate. We also wanted to control for the age difference between groups. However, as both groups differed significantly in age, the preconditions for calculating and interpreting an Analysis of Covariance were not fulfilled [33,34]. In order to nevertheless understand our data better, we calculated Spearman correlations between age and dependent variables separately for the PTSD and control group.

Reported *p* values represent exact *p* values. In addition, we report *p* values corrected for multiple comparisons with Holm’s stepwise procedure [35], applied first for the five cytokines measured in plasma and then for the three cytokines produced by PBMCs measured in presence or absence of LPS. Correlations were computed using Spearman’s rank correlation coefficient.

## Results

### Basal plasma cytokine levels

Basal plasma cytokine levels (t_1_) were generally low in both PTSD patients and controls. For TNF-α (74.6%) and IL-10 (44%), a high percentage of samples were below detection limit (BDL). Better detection rates were achieved for IL-6 (23.7% BDL), IL-8 (1.7% BDL), and MCP-1 (5.1% BDL). PTSD patients and control individuals did not differ with respect to basal plasma levels of IL-6, IL-10, TNF-α, or MCP-1; this remained true when including sex or smoking as a covariate. However, IL-8 plasma levels were significantly lower in individuals with PTSD compared to healthy control subjects (Table 3). The difference remained significant even when sex or smoking was included as a covariate, however, after correction for multiple comparisons it lost significance. For the measurements repeated after a one-week interval (t_2_), we received the same result pattern, except that the group difference for IL-8 levels was reduced to a trend [PTSD: 25% quantile 3.85, median 5.5, 75% quantile 9.08; Controls: 25% quantile 5.29, median 9.16, 75% quantile 12.71; *F* = 2.53, *p* = .08]. Nonparametric correlation analyses revealed that plasma levels for t_1_ and t_2_ were significantly related in both control and PTSD groups for IL-6 (PTSD: *r* = .85, *p* < .0001; controls: *r* = .61, *p* = .002), IL-8 (PTSD: = .76, *p* < .0001; controls: = .76, *p* < .0001), IL-10 (PTSD: *r* = .92, *p* < .0001; controls: *r* = .90, *p* < .0001), TNF-α (PTSD: *r* = .78, *p* < .0001; controls: *r* = .62, *p* = .002) and MCP-1 (PTSD: *r* = .89, *p* < .0001; controls: *r* = .74, *p* < .0001), even after controlling for multiple comparisons.

**Table 3:** Absolute numbers (cells/μl) of leukocyte subpopulations and plasma cytokine levels (pg/ml) in PTSD patients versus healthy controls

	**PTSD**			**Controls**				
**Parameter**	**25% Quantile**	**Median**	**75% Quantile**	**25% Quantile**	**Median**	**75% Quantile**	***F***	***p***
Leukocytes	5350	5820	6940	4927.5	5705	6477.5	.84	.38
Lymphocytes	1795.21	2012.1	2352.59	1611.84	1789.82	2102.555	1.77	.19
Neutrophils	2696.68	3265.82	3865.63	2696.17	2990.00	3798.70	.28	.63
Monocytes	409.74	481.28	541.13	370.13	430.44	536.72	.32	.59
Eosinophils	114.95	168.33	225.83	70.92	100.06	181.63	2.55	.12
Basophils	18.02	22.0	30.1	17.40	21.05	30.08	.09	.77
IL-6	0.2	0.5	0.9	0.07	0.35	0.73	.13	.72
IL-8	3.2	5.2	10.4	5.2	8.6	15.9	4.8	.03^#^
IL-10	0.08	0.20	0.35	0.06	0.20	1.52	2.7	.08
TNF-α	0.19	0.21	0.24	0.21	0.24	0.44	1.1	.37
MCP-1	1.15	2.00	4.05	0.83	2.30	5.95	.06	.82

* Statistical significance for differences in means of immune parameters was assessed by nonparametric permutation tests, using 10,000 random permutations of residuals under the reduced model.

# Statistical significance was lost after correction for multiple comparisons with Holm’s stepwise procedure.

### Cytokine production by PBMCs

**Figure 1:**
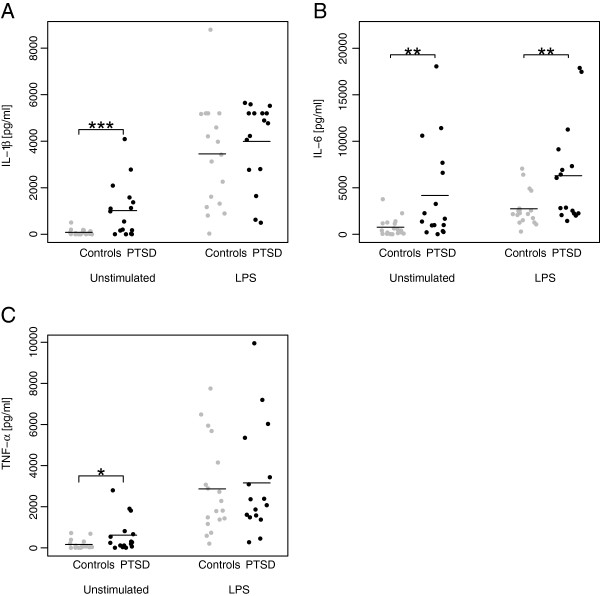
**Unstimulated spontaneous and LPS-stimulated IL-1β (A), IL-6 (B), and TNF-α (C) production by PBMCs in PTSD patients compared to controls.** The figure shows raw data, jittered horizontally to avoid overlapping points. Horizontal lines indicate means. Statistical significance for the difference in means of immune parameters was assessed by nonparametric permutation tests, using 10 000 random permutations of group labels (* p < .05, ** p < .01, *** p < .001).

Blood leukocyte distribution did not differ between PTSD patients and control individuals (Table 3), nor did it when including sex or smoking as covariates. However, isolated PBMCs from PTSD patients spontaneously produced significantly higher amounts of IL-1β (*F* = 11.31, *p* = .0003), IL-6 (*F* = 7.27, *p* = .005) and TNF-α (*F* = 5.01, *p* = .02) than PBMCs from controls (Figure 1A-C). The group differences in IL-1β, IL-6, and TNF-α production remained significant after correction for multiple comparisons. Furthermore, the findings on IL-1β and IL-6 remained when including sex or smoking as covariates, while the result for TNF-α was reduced to a trend when including smoking (*F* = 3.58, *p* = 0.06). In addition, nonparametric correlation analyses within the PTSD group revealed that PTSD symptom severity was positively correlated with the spontaneous production of IL-6 (*r* = .56, *p* = .02), and TNF-α (*r* = .58, *p* = .02), while the correlation between IL-1β and PTSD symptom severity was a trend (*r* = .47, *p* = .07). Furthermore, LPS-induced IL-6 production was significantly higher in the PTSD compared to the control group (*F* = 7.12, *p* = .01). The difference remained significant when correcting for multiple comparisons or when including sex or smoking in the model. In contrast, we could not find group differences in IL-1β and TNF-α production in LPS-stimulated PBMCs (Figure 1A-C); these latter results remained true when including sex or smoking in the analysis. To evaluate the net effect of LPS-stimulation, we further calculated the difference in cytokine production between LPS- and non-stimulated samples, but no group differences could be detected for IL-1β [PTSD: 25% quantile 1592, median 2903, 75% quantile 4339; Controls: 25% quantile 1356, median 3409; 75% quantile 5024; *F* = .35, *p* = .56], IL-6 [PTSD: 25% quantile 247, median 1053, 75% quantile 2730; Controls 25% quantile 1170, median 1461, 75% quantile 2380; *F* = .03, *p* = .87], and TNF-α [PTSD: 25% quantile 1006, median 1815, 75% quantile 3264; Controls: 25% quantile 1291, median 1938, 75% quantile 3333; *F* = .05, *p* = .84], which remained true when including sex or smoking as covariates.

### Correlations with age

Spearman correlations between age on the one hand and plasma IL-6, IL-8, IL-10, TNF-α and MCP-1 at t_1_ as well as unstimulated and LPS-induced IL-1β, IL-6 and TNF-α production by PBMCs, in each case separately for PTSD patients and control participants, were calculated. None of the correlations were significant.

## Discussion

In the present study we investigated plasma levels of IL-6, IL-8, IL-10, TNF-α and MCP-1 as well as spontaneous and LPS-stimulated *production of* IL-1β, IL-6 and TNF-α by *PBMCs* from a group of severely affected PTSD patients compared to healthy control subjects.

While *basal plasma concentrations* of pro- and anti-inflammatory cytokines did not differ significantly in PTSD patients and controls, we found evidence for a heightened inflammatory state in PTSD patients when analyzing spontaneous *ex vivo* cytokine production. Concentrations of IL-1β, IL-6, and TNF-α in supernatants of unstimulated PBMCs were significantly higher in the PTSD compared to the control group. In addition, correlation analyses within the PTSD group revealed that levels of IL-6 and TNF-α were significantly related to the severity of PTSD symptoms as assessed with the CAPS; IL-1β was also positively associated, but significance was only at the level of a trend. The group differences reported above were not ascribable to differences in blood leukocyte distribution since lymphocyte and monocyte counts were comparable in both groups. Following the introduction of smoking as covariate to the model, the finding on TNF-α was reduced to a trend. The finding of an increased spontaneous production of pro-inflammatory cytokines in the PTSD patients group suggests that PBMCs of PTSD patients are already pre-activated *in vivo*. Furthermore, as in previous studies [22,23], LPS-stimulated PBMCs from PTSD patients exhibited significantly higher IL-6 secretion compared to PBMCs from control subjects. However, when calculating the difference between LPS-stimulated and non-stimulated cytokine production to evaluate the net effect of LPS stimulation, no group differences were found, suggesting that the effect reported above mainly results from the enhanced spontaneous IL-6 secretion by PBMCs of PTSD patients. Our finding of a heightened spontaneous production of pro-inflammatory cytokines in the PTSD patient group is in agreement with reports that parameters normally suppressing inflammation are reduced in PTSD patients. For example, studies have reported lower cortisol levels [36] and a lower percentage of regulatory T cells [37] in those with PTSD, which are both crucial players in controlling inflammation thereby limiting immunopathological side effects of inflammatory processes [38,39]. Consequently our results provide further evidence for a link between PTSD and low-grade inflammation, possibly representing one psychobiological pathway from PTSD to inflammatory diseases such as arteriosclerosis and its clinical manifestations, e.g., myocardial infarction, stroke, and peripheral vascular disease, often observed in patients with PTSD [5,8]. Whereas arteriosclerosis has been formerly considered a simple lipid storage disease, current evidence supports a fundamental role for inflammation in mediating all stages of this disease. Inflammatory processes not only promote early atherogenesis and the progression of lesions, but also contribute decisively to precipitating acute thrombotic complications of atheroma. In addition, clinical studies affirm a correlation of circulating markers of inflammation with propensity to develop ischemic events and with prognosis after acute coronary syndromes [13,14].

## Conclusion

In conclusion, repeated exposure to traumatic events over the lifetime seems to induce long-lasting changes in the regulation of inflammatory processes that constitute a form of biological memory of the stressor and might lead in the long run due to self-perpetuating processes to the development of various physical diseases. However, the interpretation of our results is faced with several limitations of our study: 1) Groups differed significantly with respect to age. Unfortunately, correcting for age is only possible if groups do not differ in this variable see [33,34]. But as a) spontaneous production of IL-1β, IL-6, and TNF-α was not significantly correlated with age, neither for controls nor for PTSD subjects and b) it was positively related to PTSD symptom severity within the PTSD group, older age in the PTSD group should not have accounted for the effects reported above 2) One third of the PTSD patients took psychotropic medication. 3) We did not control for the confounding effects of physical activity on the immune outcomes. Moreover, as earlier studies suggest, a heightened inflammatory state does not seem to hold true for all studied populations of PTSD patients [20,21,24]. Future studies should therefore investigate whether a heightened inflammatory profile is associated with a specific characteristic of PTSD, e.g., whether it can only be found in PTSD patients with chronic and severe symptoms or is modulated by the type of trauma experienced. Eventually, such findings could be the basis for the development of new trauma treatment approaches, paying more attention to the potential for improving physical, in addition to mental health.

## Abbreviations

BDL: Below detection limit; CAPS: Clinician Administered PTSD Scale; CRP: C-reactive protein; HAM-D: Hamilton Depression Rating Scale; IL: Interleukin; LPS: Lipopolysaccharide; MCP: Monocyte chemotactic protein; M.I.N.I: Mini International Neuropsychiatric Interview; PBMCs: Peripheral blood mononuclear cells; PHA: Phytohemagglutinin; PTSD: Posttraumatic stress disorder; SOMS-7: Screening for Somatoform Symptoms-7; TNF: Tumor necrosis factor; WBC: White blood cell.

## Competing interests

All authors declare that they have no conflicts of interest.

## Authors’ contributions

HG, HE, MS, MG, TE, and ITK designed the study. HG, HA, and TE collected the patient data. HE, AS, MS and MG conducted the immunological analyses. HG and SK performed the statistical analyses. HG wrote the first draft of the manuscript. All authors commented on and approved the final manuscript.

## Pre-publication history

The pre-publication history for this paper can be accessed here:

http://www.biomedcentral.com/1471-244X/13/40/prepub
